# Characterization of peripheral blood inflammatory indicators and OCT imaging biological markers in diabetic retinopathy with or without nephropathy

**DOI:** 10.3389/fendo.2023.1160615

**Published:** 2023-07-03

**Authors:** Li Xiaodong, Xie Xuejun, Su Xiaojuan, He Yu, Xu Mingchao

**Affiliations:** ^1^ Department of Ophthalmology, The First Affiliated Hospital of Guizhou University of Chinese Medicine, Chengdu, China; ^2^ Department of Ophthalmology, Hospital of Chengdu University of Traditional Chinese Medicine, Chengdu, China; ^3^ Chengdu University of Traditional Chinese Medicine, Chengdu, China; ^4^ Department of Ophthalmology, Chengdu First People’s Hospital, Chengdu, China; ^5^ Traditional Chinese Medicine Hospital of Meishan, Meishan, China

**Keywords:** diabetic retinopathy, diabetic nephropathy, neutrophil-to-lymphocyte ratio (NLR), platelet-to-lymphocyte ratio (PLR), optical coherence tomography (OCT), inflammation

## Abstract

**Objective:**

To observe the distribution characteristics of peripheral blood inflammatory indexes and retinal macular area optical coherence tomography (OCT) imaging biomarkers in patients with diabetic retinopathy (DR) with or without diabetic nephropathy (DN), in order to seek clinical biomarkers that can predict the development of DR and DN.

**Methods:**

A total of 169 inpatients with DR who visited the ophthalmology department of the Affiliated Hospital of Chengdu University of Traditional Chinese Medicine from October 2020 to June 2022 and had complete clinical data were collected, and the patients with DR were divided into two major groups, DR and DR/DN, according to whether they had DN, and then further divided into four subgroups, Non-proliferative DR(NPDR), proliferative DR(PDR), NPDR/DN and PDR/DN, according to the stage of DR. The distribution characteristics of peripheral blood inflammatory indexes [Neutrophil to lymphocyte ratio(NLR) and Platelet to neutrophil ratio(PLR)], renal function indexes [Cystatin-C(CYS-C), Creatinine(Crea), Uric acid(UA)and Urinary albumin to creatinine ratio(UACR)] and OCT imaging indexes [Hyperreflective foci(HRF), Disorgnization of retinal inner layers(DRIL), Outer retinal tubulations(ORTs), Central retinal thickness(CRT), Retinal nerve fiber layer(RNFL) and Ganglion cell layer(GCL)] were analyzed between the above subgroups.

**Results:**

There was no difference between DR and DR/DN groups in terms of gender, family history of diabetes, duration of diabetes and Body mass index(BMI) (P>0.05), the mean age of the DR/DN group was significantly lower than that of the DR group (P<0.05), and the proportion of the DR/DN group with a history of hypertension was significantly higher than that of the DR group (P<0.05); there was no significant difference in hemoglobin A1C(HbA1c) between DR and DR/DN groups (P>0.05). (P>0.05), Hemoglobin(HGB) was significantly higher in the DR group than in the DR/DN group (P <0.05), NLR, PLR, Crea, UA and CYS-C were significantly higher in the DR/DN group than in the DR group (P<0.05); there was no significant difference in the comparison of HRF, DRIL, ORTs positive rate and CRT between the DR and DR/DN groups (P>0.05). RNFL and GCL thickness were significantly lower in the DR/DN group than in the DR group (P<0.05); history of hypertension (OR=2.759), NLR (OR=1.316), PLR (OR=1.009), Crea (OR=1.018), UA (OR=1.004), CYS-C (OR=3.742) were the independent (OR=0.951), age (OR=0.951), HGB (OR=0.976), RNFL (OR=0.909) and GCL (OR=0.945) were independent protective factors for DR/DN; RNFL (OR=0.899) and GCL (OR=0.935) were independent protective factors for NPDR/DN, RNFL (OR=0.852) and GCL (OR=0.928) were independent protective factors for PDR/DN. ROC curve analysis showed that the area under the curve (AUC) for CYS-C, PLR, Crea, UA and the combination of the four indicators to predict DR/DN were 0.717, 0.625, 0.647, 0.616 and 0.717, respectively.

**Conclusions:**

(1) Low age combined with hypertension HGB, NLR, PLR, CYS-C, Crea and UA may be serum biological markers for predicting DN in DR; meanwhile, PLR, CYS-C, Crea, UA and the combination of the four indicators can be used for risk assessment and adjunctive diagnosis of DN in DR combined with hypertension. (2) The RNFL and GCL thickness in the temporal aspect of the central macular sulcus may be imaging biological markers for predicting DN in DR; meanwhile, GCL thickness may have important value for risk prediction and diagnosis of DN in combination with DR.

## 1 Introduction

Diabetic retinopathy (DR) and diabetic nephropathy (DN) are common diabetic microangiopathies, and their similar pathogenesis and physiological characteristics determine their insidious onset, and they are prone to progression to irreversible visual damage and end-stage renal organopathy. Therefore, early diagnosis and timely intervention are crucial for the treatment outcome and prognosis of DR and DN. The gold standard for clinical DN diagnosis is “renal tissue biopsy”. In contrast, the diagnosis of clinical DR can be accomplished by non-invasive and simple fundus examination such as optical coherence tomography (OCT), OCT Angiography(OCTA) and fundus photography combined with clinical laboratory indicators. Studies have shown that about 26.7% of patients with type 2 diabetes have both DR and DN (1). At present, some studies have confirmed the feasibility and reliability of DR in combination with relevant clinical examinations to assist in the diagnosis of DN, such as retinal vessel curvature, diameter, branching angle and coefficient, and vascular geometry parameters such as aspect ratio (2). In addition, clinical indicators including blood lipids (3), blood bilirubin (4) and urine microalbumin (5). and urinary haptoglobin (6). and other laboratory tests. In this clinical study, we analyzed the clinical characteristics of peripheral blood inflammatory indexes, renal function indexes and retinal macular OCT biological indexes in patients with DR and DR combined with DN, explored the risk factors and protective factors of DR combined with DN, and evaluated the predictive value of DR combined with the above influencing factors for DN, in order to provide a new reference basis for the early clinical diagnosis and treatment of DN.

## 2 Research materials and methods

### 2.1 Source

this retrospective study was conducted on outpatients or inpatients with type 2 diabetes who attended the ophthalmology department of the Affiliated Hospital of Chengdu University of Chinese Medicine from October 2020 to June 2022 and had complete clinical information.

### 2.2 Inclusion criteria

(1). inpatients over 18 years of age with confirmed type 2 diabetic retinopathy with or without diabetic nephropathy.
(2). Information on the clinical consultation process, relevant laboratory tests and specialist findings of all included patients must be complete.
(3). Patients who met all the above criteria were included in this clinical study.

### 2.3 Exclusion criteria

(1). Patients with other special types of diabetes such as type 1 diabetes and gestational diabetes.
(2). Combined with severe acute complications of diabetes, such as diabetic ketoacidosis and hyperosmolar hyperglycemic states.
(3). Combined with severe organic liver and lung pathologies, cardiovascular and cerebrovascular complications, malignant tumors and hematologic diseases.
(4). Primary organic renal disease secondary to other immune, infection or drug toxicity related renal diseases, such as systemic lupus erythematosus nephritis.
(5). History of surgery in the last three months, history of acute infection or stress trauma, history of taking antibiotics, antivirals, hormones and immunosuppressive drugs that affect the laboratory results of peripheral venous blood.
(6). Combined with other ocular diseases, such as keratoconjunctivitis, glaucoma, uveitis, retinal arteriovenous obstruction, and other diseases.
(7). Those meeting any of the above may not be included in this clinical study.
(8). Ocular diseases that affect the discrimination and measurement of retinal structure levels in the macula in OCT, such as severe nuclear cataract.
(9). Extensive vitreous hemorrhage, proliferative vitreoretinal lesions in the macula anterior and macular regions of the retina, etc. (Note: This criterion is applicable to the determination and measurement of OCT biomarkers)
(10). Received vitreoretinal-related ophthalmic treatment, surgery such as vitrectomy + peel + silicone oil filling, total retinal laser photocoagulation and anti-VEGF drug vitreous cavity drug injection in the 3-6 months prior to admission. (Note: This criterion applies to the determination and measurement of OCT biomarkers)

### 2.4 Diagnostic criteria

(1). Diagnostic criteria for type 2 diabetes mellitus: according to the diagnostic criteria published by WHO (2019), the diagnosis is made by meeting any one of the following three: I. Typical diabetic symptoms (such as the presence of polyuria, polydipsia, polyphagia and unexplained weight loss) + random venous plasma glucose concentration ≥ 11.1 mmol/l; II. Fasting glucose concentration ≥7.0mmol/l (whole blood ≥6.1mmol/l); III. Two-hour glucose concentration ≥11.1mmol/l in OGTT test.
(2). Diagnostic criteria for diabetic retinopathy: I. Meet the above diagnostic criteria for type 2 diabetes and have a clear history of type 2 diabetes; II. Fundus signs refer to the international clinical grading criteria for diabetic retinopathy (as shown in **Table 1**).
(3). Diagnostic criteria for diabetic nephropathy: I. Meet the above diagnostic criteria for type 2 diabetes mellitus with a clear history of type 2 diabetes mellitus; II. According to Urinary albumin to creatinine ratio(UACR)>30mg/g and/or eGFR<60ml-min-1-(1.732)-1, and ask the nephrologist to consult to exclude other factors causing renal disease.

**Table 1:** International clinical grading criteria for diabetic retinopathy (7).

Grading	Disease severity	Lesions seen in the fundus after pupil dilatation
Level 1	No obvious retinal lesions	No abnormalities
Level 2	Mild NPDR	Microaneurysm only
Level 3	Moderate NPDR	Presence of milder than severe non-proliferative diabetic retinopathy except for microaneurysms
Level 4	Heavy NPDR	Presence of any of the following, but not yet proliferative diabetic retinopathy. a. More than 20 intraretinal hemorrhages in all four quadrants b. Venous bead-like changes in more than two quadrants c. Significant intraretinal microvascular abnormalities in more than one quadrant (IRMA sign)
Level 5	PDR	Any of the following changes occur. a. Neovascularization b. Vitreous, retinal hemorrhage or preretinal hemorrhage

## 3 Research methodology

The clinical data of all patients were collected independently by two trained ophthalmology residents through our electronic medical record system, and the fundus imaging data were collected through the database stored in Spectralis HRA+OCT by two trained ophthalmology residents who read and measured the films, and the controversial part of the data was discussed and decided with the third supervising physician.

### 3.1 General information of patients

including age, sex, weight, height, duration of diabetes and family history, history of hypertension and other general information. BMI was obtained by calculating the formula weight/height^2^ in kg/m^2^.

### 3.2 Laboratory test indexes

Hemoglobin A1C(HbA1c), Hemoglobin(HGB), Neutrophil to lymphocyte ratio(NLR), Platelet to neutrophil ratio(PLR), Cystatin-C(CYS-C), Creatinine(Crea), Uric acid(UA)and Urinary albumin to creatinine ratio(UACR). The above laboratory test results were provided by the Laboratory Department of our hospital.

### 3.3 OCT fundus imaging indexes

All patients were examined by Spectralis HRA+OCT completed by Heidelberg, Germany. Horizontal axial and vertical axial scans were performed *via* the macula centrale, and Hyperreflective foci(HRF), Disorgnization of retinal inner layers(DRIL), Outer retinal tubulations(ORTs), Central retinal thickness(CRT), and Ganglion cell layer(GCL) thickness indexes within 2000 μm from the macula centrale in the OCT images of the horizontal axial scans were selected and measured for recording. Retinal nerve fiber layer(RNFL) and GCL thickness indicators. All OCT examinations and reporting operations were performed by our attending ophthalmologist or associate ophthalmologist.

(1) HRF: isolated and well-defined borders with reflective intensity stronger than or equal to that of the RPE layer, without artifacts and with a diameter of 20-50 μm round or oval highly reflective particles are visible in each retinal layer in macular OCT (8); manual counting of HRF within 2000 μm from the central macular recess.
(2) DRIL: structural disorder or blurred borders at all levels of the inner retina visible in macular OCT (9); manual measurement of DRIL within 2000 μm from the central macular recess was determined.
(3) ORTs: the ring-shaped or oval-shaped border of highly reflective signal around the low-reflective signal located in the outer nuclear layer of the retina is visible in macular OCT (10) (2) The ORTs were identified by human measurement within 2000 μm from the central macular recess
(4) CRT: the thickness of the inner retinal boundary membrane to the RPE layer at the central recess in macular OCT; determined by the machine’s own software measurement.
(5) RNFL: the second layer of retinal hyperreflective band in macular OCT, the thickness of macular nasal hyperreflective band is thicker the closer to the optic disc, the thickness of macular temporal hyperreflective band is significantly lower than the nasal side, and the closer to the periphery, the thinner it is; RNFL thickness is determined by the machine’s own software measurement, and the thickness value at 2000 μm from the temporal side of the central macula is selected for manual correction measurement according to the specific situation.
(6) GCL: the low reflection signal band under RNFL in macular OCT; GCL thickness was determined by the machine’s own software measurement, and the manual correction measurement was performed according to the specific situation, and the thickness value at 2000 μm from the temporal side of the central macular concavity was selected.

### 3.4 Fluorescein fundus angiography examination index

FFA examination of all patients was completed by Spectralis HRA+OCT from Heidelberg, Germany, and the specific staging of DR in both eyes of patients was determined according to FFA images and reports. All patients’ FFA reports were confirmed by professors of our ophthalmology department.

### 3.5 Study grouping

All patients meeting the criteria of this study were divided into 2 major groups, DR and DR/DN, with DR group as the control group and DR/DN as the observation group; on this basis, they were further subdivided into 4 subgroups, NPDR, PDR, NPDR/DN and PDR/DN, according to the stage of DR, with NPDR as the control group and PDR, NPDR/DN and PDR/DN groups as the observation group.

### 3.6 Statistical analysis

IBM SPSS26.0 was applied for data analysis. Count data conforming to normal distribution were expressed by (x ± s), statistics by independent samples t-test, one-way ANOVA, multiple comparisons between groups by LSD method, data not conforming to normal distribution were expressed by M(Q1,Q2), multi-group statistics by Kruskal-wallis H-test, two-group and between-group Mann-Whitney was used for comparison; measurement data were statistically analyzed by chi-square test and Fisher exact test. The risk factors were analyzed by multi-factor logistic regression analysis; the ROC curves were used to analyze the critical value of each factor in predicting the level of diagnosed disease; the correlation between the factors was analyzed by Pearson correlation and Spearman correlation analysis, and *P*<0.05 indicated that the difference was statistically significant.

## 4 Results

### 4.1 Basic data

A total of 169 patients were included in this study, including 119 patients with DR and 50 patients with DR combined with DN, with an age range of 26-82 years and a mean age of 56.16 ± 10.80 years, and 96 males, accounting for 56.8% of the cases.

### 4.2 The results of peripheral blood laboratory tests in patients with DR and DR/DN in this study

#### 4.2.1 General information

As shown in **Table 2**, the proportion of hypertension history in the DR/DN group was significantly higher than that in the DR group, and the difference was statistically significant (*P*<0.05), and the mean age in the DR/DN group was significantly lower than that in the DR group, and the difference was statistically significant (*P*<0.01); although the proportion of family history of diabetes in the DR/DN group was also significantly higher than that in the DR group, the difference was not statistically significant (*P*<0.05). In addition, the differences in gender, duration of diabetes and BMI between the DR group and the DR/DN group were not statistically significant (*P*>0.05). As shown in **Table 3**, after re-grouping according to DR staging, the mean age showed a significant trend of NPDR > PDR > NPDR/DN > PDR/DN, and the difference was statistically significant (*P*< 0.01), and the proportion of hypertension history showed a trend of PDR/DN > NPDR/DN > PDR > NPDR, but the difference was not statistically significant (*P* 0.01). Their differences in gender, family history of diabetes, duration of diabetes, and BMI were not statistically significant (*P*>0.05).

**Table 2:** Comparison of general information between DR and DR/DN groups.

	DR	DR/DN	χ2/T	*P*
Male/Female (example)	63/56	33/17	2.03	0.15
Family history of DM (%)	25.21%	38%	4.83	0.18
History of HBP (%)	33.61%	52%	4.15	0.04
Duration of DM disease (years)	10 (7,18)	10 (7,16)	-0.16	0.87
BMI(Kg/m)^2^	24.27 ± 3.34	23.58 ± 2.96	1.35	0.18
Age (years)	57.8 ± 10.52	52.2 ± 10.51	3.17	0.002

**Table 3:** Comparison of general information between subgroups.

	NPDR	PDR	NPDR/DN	PDR/DN	χ2/T	*P*
Male/Female (example)	41/29	22/27	22/9	11/8	5.46	0.14
Family history of DM (%)	27.14%	22.45%	38.71%	36.84%	3.13	0.37
History of HBP (%)	31.43%	36.73%	45.16%	63.16%	6.95	0.07
Duration of DM disease (years)	10(6.5,18)	10 (7.5,17.5)	13 (10,20)	8 (6,13)	4.58	0.21
BMI(Kg/m)^2^	24.15 ± 2.78	24.44 ± 4.03	23.19 ± 2.63	24.23 ± 3.41	1.01	0.39
Age (years)	58.67 ± 10.66	56.57 ± 10.32	55.48 ± 9.82	46.95 ± 9.62	6.52	<0.001

#### 4.2.2 Laboratory indices

**Figure 1:**
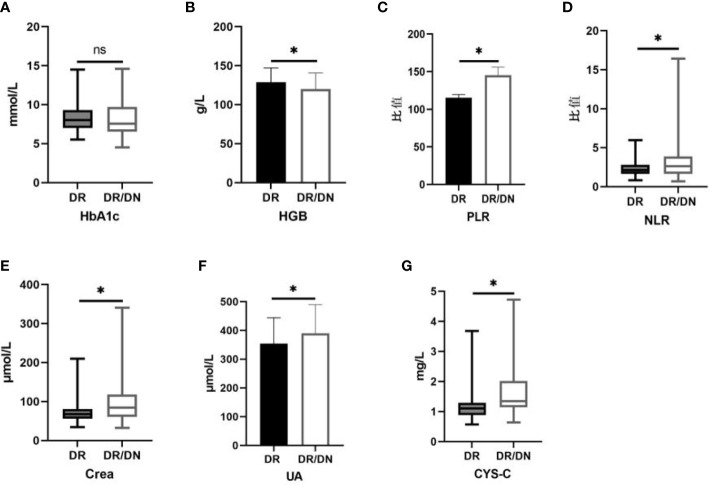
Histogram comparing laboratory test indicators between DR and DR/DN groups. (*represents *p*<0.05, indicating statistical significance). **(A-G)** Comparison of HbA1c, HGB, PLR, NLR, Crea, UA, and CYS-C results between the DR And DR/DN groups.

The results of HbA1c, HGB, NLR, PLR, Crea, UA and CYS-C in the DR and DR/DN groups are shown in **Table 4**, the level of HbA1c in the DR group was slightly higher than that in the DR/DN group, the difference was not statistically significant (*P*>0.05), the level of HGB in the DR group was significantly higher than that in the DR/DN group, the difference was statistically The differences were statistically significant (*P*<0.05), and the peripheral blood inflammatory indexes NLR and PLR and renal function indexes Crea, UA and CYS-C in the DR group were significantly lower than those in the DR/DN group, and the differences were statistically significant (*P*<0.05), as shown in **Figure 1**.

**Table 4:** Comparison of laboratory test indices between DR and DR/DN groups.

	DR	DR/DN	T/H/Z	*P*
HbA1c	8.0 (7.0,9.3)	7.5 (6.5,9.7)	-0.87	0.38
HGB	128.73 ± 18.43	120.14 ± 20.60	0.91	0.02
NLR	2.12 (1.65,2.83)	2.63 (1.64,3.88)	-1.93	0.04
PLR	115.27 ± 46.27	145.45 ± 75.02	10.134	0.002
Crea	67.60 (55.90,80.50)	84.10 (60.38,117.78)	-2.89	0.004
UA	354.03 ± 89.79	389.96 ± 99.89	-2.32	0.02
CYS-C	1.11 (0.88,1.29)	1.34 (1.14,2.01)	-4.44	<0.001

**Figure 2:**
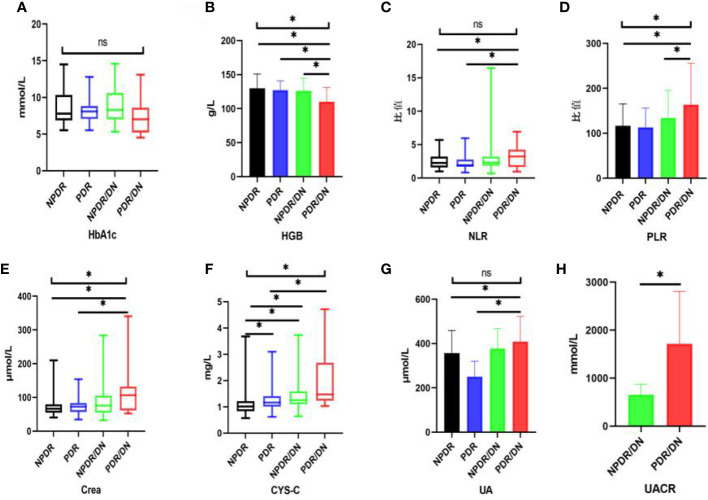
Histogram comparing laboratory test indicators between subgroups. (*represents *p*<0.05, indicating statistical significance). **(A-G)** Comparison of HbA1c, HGB, NLR, PLR, Crea, CYS-C and UA results between NPDR, PDR, NPDR/DN and PDR/DN groups; **(H)** Comparison of UACR results between NPDR/DN and PDR/DN groups.

The results of laboratory test indexes in the four groups of NPDR, PDR, NPDR/DN and PDR/DN are shown in **Table 5**, and the differences in HGB, PLR, Crea and CYS-C levels among the four groups were statistically significant (*P*<0.05). The differences between the first 3 groups and the PDR/DN group were statistically significant (*P*<0.05); PLR levels increased in the order of PDR<NPDR<NPDR< NPDR/DN<PDR/DN, and the differences between the first 2 groups and the PDR/DN group were statistically significant (*P* < 0.05); Crea and CYS-C levels increased in the order of NPDR< PDR < NPDR/DN < PDR/DN, and the differences between the first 2 groups and the PDR/DN group were statistically significant (*P*<0.05). The differences between the first two groups and the PDR/DN group were statistically significant (*P*<0.05); the levels of CYS-C increased in the order of NPDR< PDR < NPDR < NPDR/DN < PDR/DN, and the differences between the NPDR group and the other three groups and the PDR group and the PDR/DN group were statistically significant (*P*<0.05)The differences in HbA1c, NLR and UA levels between the four groups were not statistically significant (*P*>0.05), with HbA1c levels decreasing in the order of NPDR/DN > PDR > NPDR > PDR/DN, and the differences between the four groups were statistically significant (*P*> 0.05). In addition, the levels of NLR and UA increased in the order of PDR < NPDR < NPDR/DN < PDR/DN, and the differences between the first two groups were statistically significant (*P*<0.05) compared with the PDR/DN group, as shown in **Figure 2**.

**Table 5:** Comparison of laboratory test indicators between subgroups.

	NPDR	PDR	NPDR/DN	PDR/DN	T/H/Z	*P*
**HbA1c**	7.8 (6.8,10.3)	8.1 (7.0,8.8)	8.3 (7.0,10.6)	7.0 (5.2,8.6)	4.46	0.22
HGB	129.84 ± 21.12	127.14 ± 13.77	126.16 ± 18.35	110.32 ± 20.74	5.50	<0.001
NLR	2.25 (1.58, 3.23)	1.90 (1.67,2.76)	2.33(1.84,3.23)	3.23 (1.64,4.26)	4.93	0.18
PLR	116.62 ± 48.77	113.34 ± 42.87	134.29 ± 61.17	163.65 ± 92.31	4.52	0.005
Crea	66.60 (54.8,79.1)	72.40 (56.95,83.60)	75.60 (54.70,105.6)	106.1 (61.4,132.0)	13.78	0.01
UA	356.67 ± 101.96	350.24 ± 69.64	378.39 ± 89.74	408.84 ± 114.59	2.22	0.08
**CYS-C**	1.01 (0.84, 1.22)	1.16 (1.01,1.40)	1.25 (1.10,1.59)	1.47 (1.23,2.68)	31.09	<0.001
**UACR**			655.8 (143.3,871.3)	1717 (670,2805.35)	-2.57	0.01

#### 4.2.3 Factors influencing DR/DN

With DR/DN as the dependent variable and age, history of HBP, HGB, NLR, PLR, Crea, UA and CYS-C with statistically significant differences (*P*<0.05) as independent variables, the results of multifactorial logistic regression analysis showed that age and HGB were independent protective factors for DR/DN, while history of HBP, NLR, PLR, Crea, UA and CYS-C were independent risk factors for DR/DN (*P*<0.05). See **Table 6**.

**Table 6:** Risk factors and protective factors for DR/DN.

Factors	β	S.E	Waldχ2	df	*P*	OR	95% CI	
							Lower limit	Upper limit
Age	-0.50	0.017	8.861	1	0.003	0.951	0.920	0.983
History of HBP	1.015	0.369	7.556	1	0.006	2.759	1.338	5.687
CYS-C	1.320	0.350	14.213	1	<0.001	3.742	1.884	7.431
PLR	0.009	0.003	8.824	1	0.004	1.009	1.003	1.015
HGB	-0.024	0.009	6.587	1	0.010	0.976	0.959	0.994
Crea	0.018	0.005	11.220	1	0.001	1.018	1.007	1.028
UA	0.004	0.002	4.947	1	0.026	1.004	1.000	1.008
NLR	0.275	0.118	5.387	1	0.020	1.316	1.044	1.660

#### 4.2.4 Factors influencing PDR, NPDR/DN and PDR/DN

PDR, NPDR/DN and PDR/DN were used as dependent variables, age, history of HBP, HGB, NLR, PLR, Crea, UA and CYS-C were used as independent variables, and the results of multifactorial logistic regression showed that CYS-C was an independent risk factor for PDR, NPDR/DN and PDR/DN independent risk factors, and UACR was a PDR/DN independent risk factor. As shown in **Table 7**.

**Table 7:** Risk factors for PDR, NPDR/DN and PDR/DN.

Diagnosis	Influencing Factors	β	S.E	Waldχ2	df	*P*	OR	95% CI	
								Lower limit	Upper limit
PDR	CYS-C	1.755	0.764	5.271	1	0.022	5.781	1.293	25.850
NPDR/DN	CYS-C	1.829	0.806	5.144	1	0.023	6.227	1.282	30.248
PDR/DN	CYS-C	1.662	0.836	3.951	1	0.047	5.272	1.023	27.160
	UACR	0.001	0.000	9.515	1	0.002	1.001	1.000	1.002

#### 4.2.5 Laboratory tests with predictive value for DR/DN

CYS-C, PLR, Crea, and UA are meaningful for the diagnosis of DR/DN, among which the best diagnostic efficacy is CYS-C. When the value of CYS-C is greater than 1.17, the risk level of DR complicating DN increases significantly. In addition, CYS-C, Crea and UACR were significant for the diagnosis of PDR with DN, among which the best diagnostic efficacy was still CYS-C, and when the CYS-C value was greater than 1.335, the risk level of PDR with DN increased significantly. As shown in **Table 8**.

**Table 8:** Laboratory test indicators for predicting DR/DN and PDR/DN.

	Indicators	AUC	Sensitivity	Specificity	Yoden Index	Threshold value	*P*	95% CI	
								Lower limit	Upper limit
DR/DN	CYS-C	0.717	72%	65.5%	0.375	1.17	<0.001	0.628	0.805
	PLR	0.625	74%	52.2%	0.253	103.73	0.010	0.534	0.716
	Crea	0.647	52%	79%	0.31	83.55	0.003	0.546	0.767
	UA	0.616	42%	79%	0.21	406.5	0.017	0.524	0.709
	United 1	0.717	64%	72.4%	0.354		<0.001	0.629	0.804
PDR/DN	CYS-C	0.787	68.4%	76.3%	0.437	1.34	<0.001	0.687	0.866
	Crea	0.683	63.2%	71%	0.342	88.25	0.021	0.528	0.837
	UACR	0.783	57.9%	88.1%	0.43	1362.3	<0.001	0.652	0.913
	United 2	0.795	78.9%	71%	0.499		0.001	0.667	0.922

#### 4.2.6 Correlation analysis of laboratory test indicators with DR and DN staging

The results of correlation analysis showed that CYS-C, UACR, and DN staging were significantly positively correlated with DR staging (*P*<0.05), age and HGB were significantly negatively correlated with DN staging (*P*<0.05), and NLR, PLR, Crea, UA, UACR, and CYS-C were significantly positively correlated with DN staging (*P*< 0.05). In addition, the correlations between each laboratory test index are shown in **Table 9**.

**Table 9:** Correlation distribution of laboratory test indicators with DR and DN staging.

	HBP	Age	HGB	NLR	PLR	Crea	UA	UACR	CYS-C			DR	DN
HBP	1												
Age	0.142	1											
HGB	-0.140	-0.019	1										
NLR	0.214*	-0.083	-0.017	1									
PLR	0.084	-0.185	0.023	0.474*	1								
Crea	0.344*	0.137	-0.188*	0.254*	0.087	1							
UA	0.189*	0.023	-0.007	0.221*	0.018	0.498*	1						
UACR	0.485*	0.485 *	-0.466*	0.367*	0.334*	0.674*	0.432*	1					
CYS-C	0.352*	0.352 *	-0.354*	0.227*	0.161*	0.709*	0.415*	0.687*	1				
DR	0.038	-0.159*	-0.148	0.025	0.069	0.129	0.012	0.432*	0.285*			1	
DN	0.064	-0.286*	-0.324*	0.334*	0.375*	0.430*	0.297*	0.529*	0.501*			0.371*	1

*represents p < 0.05, indicating statistical significance.

### 4.3 Study the predictive value of DR for DN based on macular OCT imaging index

A total of 213 eyes of 128 patients with DR and DR/DN were included in this part, including 152 eyes with DR and 61 eyes with DR/DN, with a mean age of 55.27 ± 10.30 years and 58.59% of males.

#### 4.3.1 Comparison of OCT imaging indexes between DR and DR/DN groups

**Figure 3:**
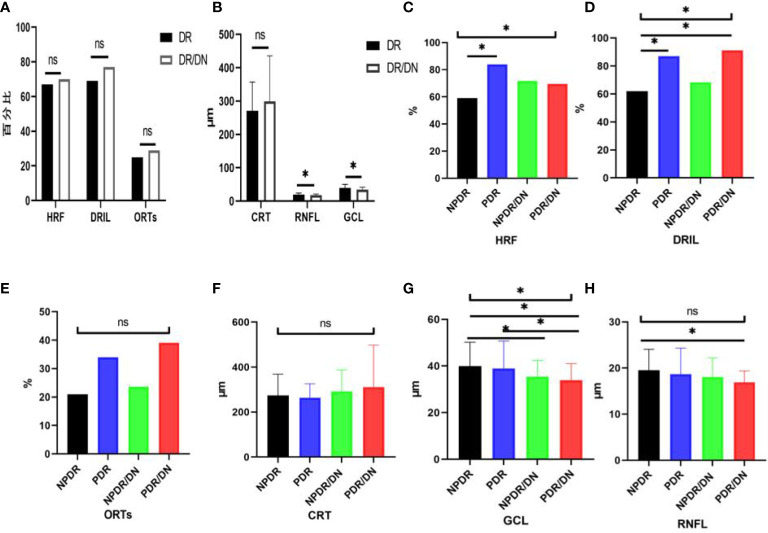
Histogram comparing DR with DR/DN and subgroup analysis of fundus imaging indices. (*represents *p*<0.05, indicating statistical significance). **(A, B)** Results of comparison of OCT indexes between DR And DN; **(C–H)** Comparison results of four OCT indexes: NPDR, PDR, NPDR/DN and PDR/DN.

As shown in **Table 10**, the positive rates of HRF, DRIL, ORTs and CRT were higher in the DR/DN group than in the DR group, but the comparative differences were not statistically significant (*P>0.05*), and the thicknesses of RNFL and GCL in the temporal aspect of the central macular sulcus were significantly lower in the DR/DN group than in the DR group, and the comparative differences were statistically significant (*P*<0.05). As shown in **Table 11**, the HRF, DRIL positivity rate and GCL thickness were significantly different between the four groups of NPDR, PDR, NPDR/DN, and PDR/DN (*P*<0.05). See **Figure 3** for details.

**Table 10:** Comparison of OCT imaging indexes between DR and DR/DN groups.

	DR	DR/DN	χ2/F	*P*
HRF	102 (67.1%)	43 (70.5%)	0.23	0.63
DRIL	106 (69.7%)	47 (77%)	1.15	0.28
ORTs	39 (25.7%)	18 (29.5%)	0.33	0.57
CRT	270.81 ± 86.31	299.00 ± 136.43	3.53	0.07
RNFL	19.26 ± 4.91	17.64 ± 3.64	5.42	0.02
GCL	39.68 ± 10.69	34.97 ± 6.97	10.53	0.001

**Table 11:** Comparison table of OCT imaging indices between subgroups.

	NPDR	PDR	NPDR/DN	PDR/DN	χ2/F	P-value
HRF	63 (59.4%)	39 (84.8%)	27 (71.7%)	16 (69.6%)	9.73	0.02
DRIL	66 (62.3%)	40 (87.0%)	26 (68.4%)	21 (91.3%)	14.53	0.002
ORTs	23 (21.7%)	16 (34.8%)	9 (23.7%)	9 (39.1%)	4.88	0.18
CRT	273.72 ± 95.11	264.11 ± 61.83	291.89 ± 95.43	310.74 ± 187.67	1.33	0.27
RNFL	19.51 ± 4.55	18.67 ± 5.67	18.08 ± 4.16	16.91 ± 2.47	2.47	0.06
GCL	39.98 ± 10.23	38.98 ± 11.76	35.42± 6.94	33.96 ± 7.07	3.70	0.01

#### 4.3.2 Fundus imaging indices of DR/DN

Multi-factor Logistic regression analysis was performed successively with DR/DN as the dependent variable and RNFL and GCL thickness as the independent variables, and the results showed that RNFL and GCL thickness were independent protective factors for DR/DN. Subgroup analysis studies were conducted with PDR, NPDR/DN and PDR/DN as dependent variables and HRF, DRIL, RNFL and GCL thickness as independent variables in multifactorial logistic regression analysis, respectively, and the results showed that HRF and DRIL were independent risk factors for PDR, while RNFL and GCL thickness were independent protective factors for NPDR/DN and PDR/DN. factors. The results are shown in **Table 12**.

**Table 12:** Results of multi-factor logistic regression analysis for DR/DN, PDR, NPDR/DN and PDR/DN.

Diagnosis	Markers	B	S.E	Waldχ2	df	*P*	OR	95% CI	
								Lower limit	Upper limit
DR/DN	RNFL	-0.095	0.037	6.753	1	0.009	0.909	0.846	0.977
	GCL	-0.056	0.017	10.580	1	0.001	0.945	0.914	0.978
PDR	HRF	1.134	0.508	4.995	1	0.025	1.322	0.119	0.870
	DRIL	1.180	0.528	4.995	1	0.025	1.307	0.109	0.865
NPDR/DN	RNFL	-0.107	0.046	5.503	1	0.019	0.899	0.821	0.983
	GCL	-0.067	0.022	9.033	1	0.003	0.935	0.896	0.977
PDR/DN	RNFL	-0.161	0.059	7.500	1	0.006	0.852	0.759	0.955
	GCL	-0.075	0.027	7.991	1	0.005	0.928	0.881	0.977

#### 4.3.3 Diagnostic value of RNFL and GCL thickness for DR/DN

GCL thickness reduction has value for the diagnosis of DR/DN, and when the GCL thickness is below 41.5 μm, the risk of DR with concurrent DN is higher; further study found that when the GCL thickness is below 42.5 μm, the risk of NPDR with concurrent DN is higher, and when the GCL thickness is below 40.5 μm, then the risk of PDR with concurrent DN is higher. RNFL thickness reduction has value for the diagnosis of PDR/DN diagnosis has some value, when RNFL thickness is lower than 20.5 μm, then PDR and found DN risk is higher. See **Table 13**.

**Table 13:** Prediction data table of RNFL and GCL for DR/DN, NPDR/DN, PDR/DN.

	Indicators	AUC	Sensitivity	Specificity	Yoden Index	Threshold value	*P*	95% CI	
								Lower limit	Upper limit
DR/DN	RNFL	0.579	80.3%	36.8%	0.171	20.5	0.07	0.500	0.659
	GCL	0.642	85.2%	48.0%	0.332	41.5	0.001	0.569	0.716
NPDR/DN	RNFL	0.522	97.4%	56.0%	0.134	24.5	0.678	0.423	0.620
	GCL	0.605	86.8%	40.4%	0.262	42.5	0.043	0.521	0.689
PDR/DN	RNFL	0.636	95.7%	36.3%	0.31	20.5	0.034	0.546	0.725
	GCL	0.642	91.3%	45.3%	0.366	40.5	0.026	0.546	0.738

#### 4.3.4 Correlation between OCT imaging indices and DR and DN staging

The results of correlation analysis showed that HRF, ORTs, DRIL, and DN staging were significantly positively correlated with DR staging (*P*<0.05), and no significant correlation was seen between each imaging index and DN staging (*P*>0.05), and the correlation between each imaging index is shown in **Table 14**.

**Table 14:** Correlation distribution of OCT imaging indices with DR and DN staging.

	HRF	CRT	ORTs	RNFL	GCL	DRIL	DR	DN
HRF	1							
CRT	0.361*	1						
ORTs	0.391*	0.427*	1					
RNFL	-0.041	0.034	-0.096	1				
GCL	0.047	0.005	-0.128	-0.149*	1			
DRIL	0.467*	0.237*	0.331*	0.018	0.063	1		
DR	0.315*	0.091	0.228*	-0.090	-0.031	0.438*	1	
DN	0.116	-0.184	0.102	0.011	-0.205	0.047	0.334*	1

*represents p<0.05, indicating statistical significance.

## 5 Discussion

### 5.1 Young age and hypertension are risk factors for DR Complicated with DN

This study showed that the mean age of patients in the DR/DN group was significantly lower than that of the DR group, and subgroup analysis showed that the mean age of the PDR/DN group was below 50 years old, and the age of the PDR, NPDR/DN and PDR/DN groups showed a significant trend toward younger age compared with the NPDR group, and the results of multifactorial analysis indicated that age was a protective factor for DR/DN. A national study (11) also found that the age of the PDR patient group was significantly younger than that of the NPDR patient group, In the study of Cheng Haihai (12), it was shown that younger DN patients were more likely to have DR complications (12), which is also consistent with the results of the present study. The above findings suggest that older DR patients have a lower risk of DN complications and younger DR patients have a higher risk of DN complications. Although age is an uncontrollable factor, it also suggests that more clinical caution should be taken in younger DR patients, and more frequent outpatient follow-up is needed to intervene in time for the possible onset or progression of DN. In addition, the proportion of the DR/DN group with a history of hypertension was found to be significantly higher than that of the DR group in this study, and the history of hypertension was an independent risk factor for DR/DN, which is basically consistent with domestic and international studies, in which Pu Danfeng ‘s (13) study found that the duration of hypertension and systolic blood pressure were independent risk factors for DR/DN, and the other study (14) also confirmed that hypertension is indeed closely associated with the progression of DR and DN. Therefore, strict control of hypertension can significantly reduce the risk of DR and DN development.

### 5.2 High HGB level is a protective factor for DR Concurrent DN

Hyperglycemia is a controllable risk factor for diabetic complications. Chinese Guidelines for the Prevention and Treatment of Type 2 Diabetes (15) HbA1c has become a new criterion for the diagnosis of diabetes and an important indicator for predicting chronic microvascular complications of diabetes. When HbA1c is greater than 7%, the risk of developing DR and DN is significantly higher, and the HbA1c in both the DR and DR/DN groups in this study was significantly greater than 7%. It has also been confirmed in previous studies that HbA1c is an important indicator of DR (11) and DN (16). In the present study, HbA1c was lower in the DR/DN group than in the DR group and was significantly lower in the PDR/DN group than in the NPDR/DN group, but the difference between the groups was not statistically significant. We speculate that it may be related to the fact that patients with DR/DN, especially PDR/DN, strictly control their blood glucose level after the symptoms of renal dysfunction or after the obvious loss of vision, while patients with NPDR alone tend to ignore their ocular symptoms and do not pay attention to them or do not take regular glucose-lowering treatment, so the level of blood glucose control is poor; meanwhile, HbA1c is a product of HGB binding to glucose in red blood cells, so its At the same time, HbA1c is the product of HGB binding to glucose in erythrocytes, so its decrease is not only related to blood glucose but may also be closely related to the change of HGB concentration in the same period. The HGB in the DR/DN group in this study was significantly lower than that in the DR group, and the mean HGB values between subgroups still differed significantly, and the mean HGB values in the PDR/DN group were significantly lower than those in the other 3 groups, which is also basically in line with the trend of HbA1c result changes in this study. In addition, the results of this study found that HGB was a protective factor for DR/DN. Results of a Korean cross-sectional study based on 2123 patients with diabetes mellitus (17) showed that high HGB levels significantly reduced the risk of DR prevalence; in addition there are also scholars (18) found a significant negative association between HGB levels and the index and severity of retinal ischemia in DR; Previous studies (19) have also confirmed that anemia is an independent risk factor for DR and DN; combined with the above studies, high HGB levels have been shown to delay the development of DR and DN. Therefore, we speculate that HbA1c and HGB are mutually influential factors, suggesting that the HGB of patients with DR and DN should be closely monitored in clinical practice, and combined with fasting and postprandial glucose, the true glycemic control level of patients should be comprehensively evaluated.

### 5.3 PLR and NLR may be peripheral blood inflammatory biomarkers of DN predicted by DR

NLR and PLR are novel non-specific inflammatory markers that have received more attention in recent years. elevated levels of NLR and PLR represent an increase in neutrophils and platelets, which release large amounts of inflammatory cytokines that inhibit lymphocyte inflammatory regulation and protection, and the immune balance between neutrophils and lymphocytes is disrupted (20), with tumors (21), cardiovascular and cerebrovascular complications (19), respiratory diseases (22), diabetes mellitus (23) and other systemic chronic inflammatory diseases are closely related, NLR value is independently associated with neovascular Age-related macular degeneration (24). Moreover, Higher PLR could reduce the risk of Type 2 Diabetes Mellitus. Larger increase of NLR could increase Type 2 Diabetes Mellitus risk (25). And an increasing number of scholars are now studying their relationship with diabetic microvascular complications. One study showed that (26) Platelets and neutrophils interact with each other, and under inflammatory stimulation neutrophils produce platelet chemokines and activating factors, and activated platelets release large amounts of pro-inflammatory and pro-angiogenic factors that damage vascular endothelial cells, leading to increased vascular permeability, edema, and exudation, which further activate neutrophils to release more inflammatory mediators and induce chronic microangiopathy and inflammatory responses (26), promoting DN (27) and DR (28) of progression. Recent findings suggest that Lymphocyte percentage can be used as an inflammatory marker for the development of DME in patients with severe DR (29).The NLR and PLR in the DR/DN group in this study were significantly higher than those in the DR group, suggesting a more severe inflammatory response in DR/DN; previous studies also found that NLR predicted DN progression (30) and the severity of DR (31); a recent study (32) demonstrated that NLR and PLR are risk factors for DN and may be predictors of early DN patients (33); another study (34) also showed that NLR was an independent risk factor for DR, DN, and DR/DN, Latest Research suggest that PLR may be an independent risk factor for evaluating DR in type 2 diabetes patients (35).The present study found that both NLR and PLR were independent risk factors for DR/DN, and the risk of DR complicated by DN was significantly increased when PLR > 103.73. Previous studies found that PLR also predicted diabetes-related lower extremity vascular disease (36), atherosclerosis and diabetic foot ulcers (37). This suggests that platelets are not only involved in thrombosis, but also play an important role in regulating the immune inflammatory response, especially in the intravascular one. Meanwhile, correlation analysis showed that PLR was significantly positively correlated with NLR, and both PLR and NLR were significantly positively correlated with DN stage; Furthermore, The systemic immune-inflammation index (SII) is a novel and integrated inflammatory biomarker, SII was calculated as the platelet count × neutrophil count/lymphocyte count. New research results indicate that the higher SII level is associated with DN in Type 2 Diabetes Mellitus patients. The SII could be a cost-effective and straightforward approach to detecting DN (38). In conclusion, we speculate that PLR and NLR are important inflammatory biomarkers for predicting DN in DR, and are closely related to DN severity.

### 5.4 CYS-C, UA and Crea may be peripheral blood biomarkers of DN predicted by DR

2. Serum CYS-C, an endogenous marker of glomerular filtration rate, is associated with diabetic macroangiopathy (39) and microangiopathy (40) both have relevance. In the present study, CYS-C was significantly higher in the DR/DN group than in the DR group, and CYS-C was an independent risk factor not only for DR/DN but also for PDR, NPDR/DN and PDR/DN, respectively. Previous clinical studies (41) found that Cys C was an independent risk factor for DN complicated by DR; the present study confirmed that CYS-C had predictive value for DR/DN and PDR/DN, and the likelihood of DR complicated by DN was higher when CYS-C> 1.17 mg/L, and the risk of PDR complicated by DN was higher when CYS-C was elevated to 1.335 mg/L. Other clinical studies (42)have found a significantly increased risk of renal insufficiency in PDR patients with CYS-C above 1.315 mg/L. The most recent Meta-analysis (43) The results showed that CYS-C has significant diagnostic value for DN, and the abnormal rate of CYS-C in the urine of patients gradually increased with the aggravation of DN, and the abnormal rate of CYS-C in the urine of patients with stage 3 DN was 100% (44); and our study showed a significant positive correlation between serum CYS-C and DN stage. One study showed that CYS-C could be an early predictor of tubular injury in T2DM diabetic patients (45).A domestic study (34) found that CYS-C was only associated with DN and weakly correlated with DR, which may be related to the fact that the study did not group the severity of DR.Recent research has discovered that CYS-C was a risk factor for DN independent of BMI and SBP in diabetes mellitus patients (46). Zheng Yanhui et al (47) found that CYS-C was an independent risk factor for DR and positively correlated with the severity of DR, while the present study also found that CYS-C was an independent risk factor for PDR and significantly and positively correlated with the stage of DR. A Meta (48) analyzed the correlation between CYS-C and DR in 4354 Chinese patients with type 2 diabetes and confirmed that CYS-C was strongly associated with DR progression, which is consistent with the results of the present study. In addition, one study found that CYS-C promotes increased VEGF levels to induce angiogenesis (49) and was closely associated with the expression of various inflammatory factors CRP, IL-6 and TNF-α (50), CYS-C levels were significantly positively correlated with PLR and NLR in the present study, suggesting that CYS-C levels may be related to the degree of inflammatory response *in vivo*. Therefore, CYS-C can be used as an important serum indicator for predicting the development of DN in DR.

Serum UA is the end product of purine degradation and has antioxidant and pro-oxidant properties, UA can decrease glucose-stimulated insulin secretion and cause β-cell death. The mechanisms underlying these effects are UA-induced oxidative stress and inflammation within the β-cells. UA also stimulates inducible nitric oxide (NO) synthase (iNOS) gene expression leading to NO-induced β-cell dysfunction. Thus hyperuricemia may potentially cause β-cell dysfunction, leading to diabetes (51). High levels of UA stimulate proliferation of human vascular smooth muscle cells, increase the expression of endothelin-1 and CRP, increase ROS production, and damage vascular endothelial cells (52), leading to macrovascular and microangiopathy. Therefore, excessive UA is strongly associated with diabetic microangiopathy (53). In this study, UA was significantly higher in the DR/DN group than in the DR group, and UA was an independent risk factor for DR/DN, and the risk of DR complicating DN increased when UA was >406.5 μmol/L; Lai Na’s study (54) showed that the risk of DR occurred significantly increased when UA exceeded 304.75 μmol/L, while the risk of DN occurred significantly increased when UA > 379.05 μmol/L (54). The above studies suggest that high UA can be a better predictor of the development of DR and DN. In the correlation analysis, UA levels were significantly and positively correlated with DN staging, which is consistent with previous studies (55). In addition, scholars (56) found that UA levels increased with increasing DR severity and could be used as a clinical indicator to assess the severity of DR, and that men with high UA had a significantly higher risk of developing DR (57). However, the results of this study showed no significant correlation between UA and DR staging, and the difference in UA comparison between the DR and PDR groups in the subgroup analysis was not statistically significant, which may be related to the small sample size included in this study. There is a relevant study found that higher UA levels are associated with various stages of the onset and progression of DN, including metabolic, cardiovascular and kidney function abnormalities (58).Therefore, serum UA may be a marker for DR to predict DN and assess the severity of DN.

Currently, although Crea is affected by various factors such as age, gender and weight, it is also one of the most direct markers for clinicians to determine renal function, and an increase in Crea indicates a decrease in glomerular filtration. The present study found that Crea is an independent risk factor for DR/DN, and the likelihood of DN complicating DR increases when Crea>83.55μmol/L, and the risk of DN complicating PDR increases when Crea rises to 88.25 μmol/L. The significant positive correlation between Crea level and DN stage suggests that Crea can be a serum marker for assessing the severity of DN and predicting DN by DR. This suggests that Crea can be a serum marker to assess the severity of DN and to predict DN in DR. Previous studies have also confirmed that regular monitoring of Crea levels is useful in assessing the progression and prognosis of DN (59, 60). In addition, the Crea levels in the PDR group were found to be more than adequate. In addition, Crea levels were higher in the PDR group than in the DR group, but the difference was not statistically significant and did not correlate with DR staging, suggesting that Crea has limited value in assessing DR severity. According to the results of this study, Crea is a helpful serum marker for predicting DN in DR.

### 5.5 UACR may be a biomarker of DN predicted by DR

UACR is an effective indicator for monitoring early proteinuria and chronic renal impairment. In this study, we found that UACR is an independent risk factor for PDR/DN and has diagnostic significance for PDR/DN, and when the combination of UACR, CYS-C and Crea can improve the predictive value of PDR/DN, and all of them are higher than the diagnostic value of each individual index. Gao Jun et al. (61) found that the combination of UACR, serum Cys-C and amyloid A had a higher diagnostic value for early DN than each individual index, and could be used as a clinical index for monitoring early DN. In this study, UACR was significantly and positively correlated with DN staging, and we hypothesized that UACR could be used to assess the severity of DN. In addition, UACR is also a risk factor for the development of DR (62)., study (63) suggests that high values of UACR within the normal range may be a risk factor for the onset of DR, and the likelihood of DR onset is higher when UACR reaches 16.79 mmol/L. In this study, the UACR index was not collected from patients with DR alone, so it could not be verified whether UACR was a risk factor for DR onset, but correlation analysis found that it was closely related to the staging of DR, indicating that the higher the UACR, the more severe the DR. In conclusion, UACR may be a clinical marker for DR to predict the progression of DN with the aid of diagnosing DN complicated by PDR.

### 5.6 HRF, DRIL, ORTs and CRT have limited value for DR Prediction DN

At present, macular OCT biological markers such as HRF, DRIL, ORTs and CRT are mainly used to evaluate and monitor the changes of DME condition, treatment effect and visual prognosis. This study mainly analyzed the distribution characteristics of the above OCT biological markers in DR and DR/DN patients. The presence of HRF in all layers of the retina suggests active inflammation of the retina and even the choroid, and more scholars believe that its formation may be related to the activation of microglia and the accumulation of pro-inflammatory factors released, among others (64). DRIL represents a signal disruption and disruption of the second level neurons of the visual transmission pathway and may be closely related to retinal ischemia and hypoxia, previous studies (65) found a significant correlation between DRIL and the area of the retinal nonperfused area, and also studies (66) DRIL and its length are also important biological predictors of DR prognosis The DRIL and its length are also important biological predictors of DR prognosis (67).We found that the positive rates of HRF and DRIL were higher in the DR/DN group than in the DR group, but not statistically significant, and in the subgroup analysis, the positive rates of HRF and DRIL were significantly higher in the PDR group than in the NPDR group, and HRF and DRIL were independent risk factors for PDR, and the positive rates of HRF and DRIL were also significantly and positively correlated with DR staging, but they did not correlate with either DN staging; the above The results suggest that HRF and DRIL can be biological markers for predicting PDR and DR staging, but have limited predictive value for DN. ORTs mostly occur in the outer nuclear layer of the retina in the macula, and its formation may be associated with the remodeling of photoreceptor cells after retinal damage, and the progression of retinal lesions to the end stage (10).ORTs are suggestive for the progression and treatment prognosis of a variety of CNV-related and degenerative genetic-related retinal diseases, and are used to predict sensitivity to anti-VEGF drugs and visual prognosis, but not as a basis for assessing CNV activity and retreatment (68). In this study, the positive rate of ORTs was found to be significantly correlated with DR staging and not significantly correlated with DN staging.CRT is a common index to assess the indication, efficacy and prognosis of drug therapy for a variety of macular edema diseases and is widely used in clinical and basic research of various retinal and choroidal diseases. In this study, CRT was significantly higher in the DR/DN group than in the DR group, but it was not statistically significant, and no significant correlation was found between CRT and the staging of DR and DN. This may be because DME can occur in all stages of DR, which affects the variability of CRT between groups, or it may be related to the inclusion of more patients with moderate to severe NPDR and PDR in this study.

### 5.7 RNFL and GCL thickness in macular area may be biomarkers of OCT image for DR Prediction of DN

The current findings mostly support that neurodegenerative changes in the retina have occurred in DR prior to the development of microangiopathy, leading to neuronal apoptosis and glial cell activation, which mainly affects retinal ganglion cells (RGCs) in the RNFL, GCL, and inner plexiform layer, which contains the axons of RGCs, and the GCL and inner plexiform layer, which consists of the nuclei and dendrites of RGCs (69), so the measurement of RNFL and GCL thickness can provide some reference for neurodegenerative changes in the retina. In this study, RNFL and GCL thicknesses were significantly lower in the DR/DN group than in the DR group, and RNFL and GCL thicknesses were independent protective factors for DR/DN, NPDR/DN, and PDR/DN. When RNFL thickness was <20.5 μm, it was diagnostic for PDR/DN, and GCL thicknesses <41.5 μm, 42.5 μm, and 40.5 μm were diagnostic for DR/DN,The above results suggest that RNFL and GCL thickness can be used as biological markers of DR to predict DN, and also confirm the predictive value of retinal neurodegenerative lesions for DN. In previous domestic and international studies, there are relatively few studies related to the thickness changes of RNFL and GCL in the macula at the same time, and most of them study DR from the changes of RNFL thickness around the optic disc (70), open-angle glaucoma (71), myopia (72) and other early neurodegenerative changes in optic nerve and macular diseases, so these studies are not very comparable to the results of the present study. Wang Lili et al (73) found that the thickness of RNFL in the macula has become significantly thinner in diabetic patients compared with normal subjects, so regular monitoring of thickness changes using OCT can help in the early detection of diabetes and early diagnosis and treatment of DR (74).. In the subgroup analysis of this study, the RNFL thickness in the PDR group was lower than that in the NPDR group, but it was not statistically significant, and no significant correlation was found between RNFL thickness changes and DR staging, which may be related to the fact that a normal control group was not established in this study. In addition, Diao Lili et al (75) found a significant thinning of retinal GCL thickness in the macula of diabetic patients, but there was no significant relationship between its thickness change and DR severity, which is consistent with the result that there was no significant correlation between GCL thickness and DR staging in the present study, and is also consistent with Chhablani et al (69) study conclusions. In conclusion, the temporal RNFL and GCL thickness in the macula may be OCT imaging biological markers of DR to predict DN.

## 6 Conclude

In this part of the study, we found that low age, hypertension, hyperglycemia, anemia, increased inflammatory response, impaired renal function and retinal neurodegenerative changes were important risk factors for DR complicated by DN from an ophthalmic perspective by analyzing the general data, distribution characteristics of laboratory and fundus OCT imaging indices in patients with DR and DR complicated by DN. Inflammatory indicators PLR and NLR, renal function indicators CYS-C, Crea and UA, and OCT imaging indicators macular temporal RNFL and GCL thickness may be important clinicobiological markers for predicting DN complicated by DR when PLR > 103.73, CYS-C>1.17 mg/L, UA > 406.5 μmol/L, Crea > 83.55 μmol/L, RNFL thickness <20.5 μm and GCL thickness <41.5 μm, the risk of DR complicating DN was significantly increased, and the combination of PLR, CYS-C, UA and Crea did not improve the diagnostic value of DR complicating DN; when CYS-C >1.335 mg/L, Crea >88.25 μmol/L, and The combination of CYS-C, Crea and UACR significantly increased the diagnostic value of PDR with DN. The positive correlation between DR staging and DN staging indicated that DR and DN were closely related, and the two were predictors of each other. We speculate that the consistency of the association and progression of DR and DN may be related to the inflammatory response as their common pathogenesis. The inflammatory response of the renal tissue leads to impaired glomerular filtration function, therefore proteinuria, abnormal elevation of clinical serum inflammatory indexes and renal function indexes; the inflammatory response of the retinal tissue leads to retinal microangiopathy and neurodegenerative changes, and macular OCT reveals HRF, ORTs, and DRIL and thinning of RNFL and GCL thickness were seen on macular OCT.

## Data availability statement

The raw data supporting the conclusions of this article will be made available by the authors, without undue reservation.

## Ethics statement

Ethical review and approval was not required for the study on human participants in accordance with the local legislation and institutional requirements. Written informed consent for participation was not required for this study in accordance with the national legislation and the institutional requirements.

## Author contributions

LX designed the study and drafted the manuscript as the first author. SX and LX carried out the literature search. HY contributed to data extraction and quality assessment. XX and XM supervised the study and XM as the corresponding author. All authors contributed to the article and approved the submitted version.
